# Optimization and Standardization of Plant-Derived Vascular Scaffolds

**DOI:** 10.3390/ijms26062752

**Published:** 2025-03-19

**Authors:** Gianna Imeidopf, Dara Khaimov, Sashane John, Nick Merna

**Affiliations:** 1 Fred DeMatteis School of Engineering and Applied Science, Hofstra University, Hempstead, NY 11549, USA; gimeidopf1@pride.hofstra.edu (G.I.); dkhaimov2@pride.hofstra.edu (D.K.); sjohn2@pride.hofstra.edu (S.J.); 2 Cardiothoracic Surgery, Northwell Health, New York, NY 10022, USA

**Keywords:** plant-derived scaffolds, decellularization, tissue engineering, vascular grafts

## Abstract

Vascular graft failure rates remain unacceptably high due to thrombosis and poor integration, necessitating innovative solutions. This study optimized plant-derived extracellular matrix scaffolds as a scalable and biocompatible alternative to synthetic grafts and autologous vessels. We refined decellularization protocols to achieve >95% DNA removal while preserving mechanical properties comparable to native vessels, significantly enhancing endothelial cell seeding. Leatherleaf viburnum leaves were decellularized using sodium dodecyl sulfate-based and Trypsin/Tergitol-based treatments, achieved via clearing in bleach and Triton X-100 for 6 to 72 h. To assess the environmental influence on scaffold performance, leaves from multiple collection sites were processed using sodium dodecyl sulfate-based protocols. Scaffold performance was evaluated through tensile testing and histological analysis to assess structural integrity, while DNA quantification and endothelial cell recellularization measured biological compatibility. Sodium dodecyl sulfate-treated scaffolds with shorter clearing durations demonstrated the highest DNA removal (≥95%) while preserving mechanical properties, significantly outperforming Trypsin/Tergitol treatments. Longer clearing times reduced fiber diameter by 60%, compromising scaffold strength. Shorter clearing times preserved extracellular matrix integrity and significantly improved endothelial cell seeding efficiency. Larger leaves supported significantly higher endothelial cell densities than smaller leaves, highlighting the need for standardized material sources. Permeability tests demonstrated minimal leakage at 120 mmHg and structural stability under dynamic flow conditions, suggesting their suitability for vascular applications. These findings establish a reliable framework for optimizing plant-derived grafts, improving their reproducibility and performance for tissue engineering applications.

## 1. Introduction

Despite decades of innovation, vascular graft failure remains a major barrier to effective cardiovascular treatment, leading to high rates of reoperation and mortality [1,2,3]. Autologous vessels, including the saphenous vein, are the gold standard for small-diameter vascular grafts, but availability is limited due to patient-specific factors [4]. Synthetic alternatives, such as expanded polytetrafluoroethylene (ePTFE) and Dacron, have been developed but exhibit poor long-term patency due to thrombosis, compliance mismatch, and intimal hyperplasia [5,6]. Hybrid approaches combining synthetic and biological materials have attempted to address these limitations, but challenges, such as manufacturing complexity, suboptimal biocompatibility, and high production costs, persist [7,8,9].

Decellularized plant-derived scaffolds provide a biocompatible, cost-effective alternative with a cellulose-based matrix that supports endothelialization and offers tunable mechanical properties [10,11,12,13]. For instance, Modulevsky et al. have demonstrated that decellularized apple-derived cellulose scaffolds, when implanted subcutaneously in mice, elicited a transient foreign body response that subsided over eight weeks, with subsequent fibroblast infiltration and collagen deposition, indicating favorable biocompatibility [14]. Plant-based scaffolds mimic the structural and mechanical characteristics of native tissues, promoting cellular attachment, and have demonstrated potential for use in skeletal, cardiac, and vascular applications [14,15,16,17]. Decellularization removes cells while preserving the extracellular matrix (ECM), enabling recellularization with mammalian cells. These protocols often include the use of clearing solutions, which contain agents such as bleach and Triton X-100, to enhance optical transparency and facilitate cellular removal [18,19]. The decellularization protocol used and the source of plant tissue highly influence the structural integrity of the ECM and its compatibility with seeded cells [20,21]. These factors must be optimized to ensure the reproducibility and functionality of the scaffolds [22]. Here, we optimized a plant-derived scaffold that achieved high endothelial cell (EC) seeding efficiency while preserving mechanical integrity, providing a standardized approach to vascular tissue engineering. By refining decellularization protocols and evaluating material source variability, we establish a framework for improving graft reproducibility and performance, addressing key barriers to broader tissue engineering applications.

Permeability is a fundamental property of vascular grafts, influencing both short-term surgical handling and long-term biological integration. In synthetic grafts, permeability is typically controlled through material selection, pore size engineering, and surface coatings to regulate fluid flow and cellular infiltration. Uncoated knitted Dacron grafts exhibit high permeability, necessitating preclotting or external sealing to prevent perioperative bleeding, while ePTFE grafts achieve controlled permeability by fine-tuning their internodal microstructure [23]. In plant-derived scaffolds, permeability is dictated by the decellularization process, the intrinsic porosity of the plant matrix, and subsequent modifications. Excessive permeability may lead to immediate graft leakage, whereas insufficient permeability can hinder nutrient diffusion and cellular infiltration, both of which are critical for remodeling and integration. Recent studies have utilized dye-based perfusion tests to visually assess permeability in decellularized plant vasculature, confirming open microchannels with some minor leakage [10]. However, no studies have investigated permeability across the leaf’s lamina for vascular tissue engineering applications, leaving a gap in understanding with regard to how fluid transport occurs through the scaffold’s structure beyond the primary vascular channels.

While previous studies have demonstrated the feasibility of plant-derived scaffolds for tissue engineering, critical gaps remain that hinder their clinical translation. Existing decellularization methods often compromise plant structure, leading to increased porosity and decreased mechanical properties [12]. Another key issue is the lack of standardization in decellularization protocols and material sourcing, which impacts reproducibility and scalability [13]. Decellularization parameters (detergent composition and clearing durations) influence mechanical strength and ECM integrity, which in turn affect recellularization potential. However, their effects on plant-derived ECM remain poorly understood [20,24]. Additionally, variations in plant material due to environmental conditions, such as sunlight exposure, soil composition, and leaf morphology, may introduce inconsistencies in scaffold production that hinder scalability and clinical application [25,26]. Addressing these gaps is essential for developing standardized, reproducible protocols for plant-based vascular grafts.

Leatherleaf viburnum (*Viburnum rhytidophyllum*) was selected as the model plant species for this study based on prior studies demonstrating its superior mechanical integrity [11,24]. Compared with other plant-derived scaffolds, such as spinach and parsley, leatherleaf viburnum exhibits a more robust ECM structure, which maintains tensile strength and elasticity after decellularization, closely matching the mechanical properties of native blood vessels. Specifically, the tensile strength of leatherleaf viburnum (0.24 ± 0.05 N/mm^2^) is significantly higher than that of spinach (0.07 ± 0.02 N/mm^2^) and parsley (0.10 ± 0.04 N/mm^2^). This enhances the ability of the graft to withstand physiological forces of blood flow. Furthermore, suture retention of our leatherleaf grafts met transplantation standards, with retention forces of 0.74 ± 0.13 N and 0.65 ± 0.28 N, compared with 1.01 ± 0.17 N and 0.32 ± 0.10 N for rat aorta when using 8–0 and 10–0 Prolene sutures, respectively [27,28,29]. This minimizes the risk of tearing during suturing and implantation. Lastly, the larger leaf size of leatherleaf enables fabrication of small-diameter grafts of physiologically relevant dimensions, unlike smaller leaves such as spinach and parsley, which are more limited in scale. Other commonly decellularized plant materials, such as apple, celery, bamboo, and leek, were also considered but did not meet our criteria for vascular applications [30,31,32].

This study optimized plant-derived ECM scaffolds for vascular tissue engineering, focusing on the interplay between decellularization protocols and material sources. Using leatherleaf viburnum as a model system, we evaluated the effects of different decellularization methods and clearing durations on DNA removal, mechanical properties, and ECM structure. We also examined how variations in four leaf collection sites influenced scaffold performance, emphasizing the need for standardization. Sodium dodecyl sulfate (SDS)-treated scaffolds with shorter clearing durations emerged as the most effective, achieving high DNA removal efficiency while preserving mechanical properties and ECM structure, which facilitated superior EC seeding. These findings underscore the critical role of protocol optimization and material standardization in advancing plant-based vascular grafts for clinical applications.

## 2. Results

### 2.1. Decellularization Efficiency

**Figure 1:**
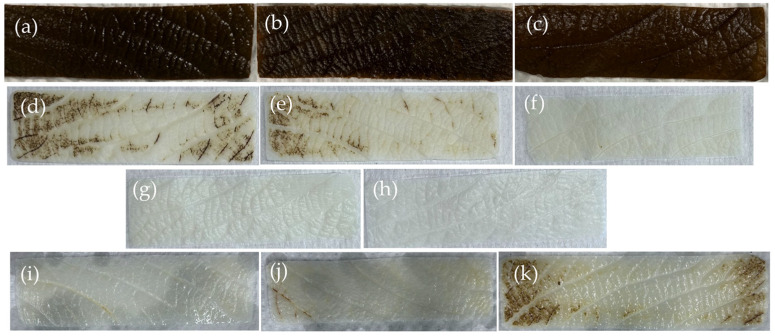
Representative images of (**a**) Trypsin/Tergitol-, (**b**) Trypsin/Tergitol/EGTA- and (**c**) SDS/Tergitol-decellularized leatherleaf viburnum; SDS-decellularized leatherleaf with (**d**–**h**) 6, 12, 24, 48, and 72 h clearing using Location 1, respectively; and SDS-decellularized leatherleaf with 6 h clearing using (**i**–**k**) Locations 2, 3 and 4, respectively.

SDS with clearing solution removed 99% of DNA from leatherleaf, significantly outperforming Trypsin/Tergitol treatments in DNA removal. SDS/Tergitol-treated samples had the lowest DNA removal (52%), while SDS and clearing solution progressively reduced DNA content with longer clearing times (95–99% removal). SDS-treated samples appeared opaque and whitish, while other treatments produced a greenish-brown hue (Figure 1). To assess variability, we also evaluated decellularization efficiency across four leatherleaf collection sites (Locations 1–4), which differed in environmental conditions. Small differences in decellularization efficiency were observed between these locations. DNA content (ng/mg tissue) was 280 ± 147, 27 ± 4, 37 ± 34, 18 ± 16, 50 ± 11 for non-decellularized leatherleaf and leatherleaf from Locations 1, 2, 3, 4, respectively. Residual DNA in SDS-decellularized leatherleaf represented satisfactory decellularization efficiency (<50 ng DNA/mg tissue) [33]. Having established that SDS-treated scaffolds achieve the highest DNA removal efficiency, we next evaluated their mechanical properties to determine their suitability for vascular graft applications.

### 2.2. Mechanical Properties

**Figure 2:**
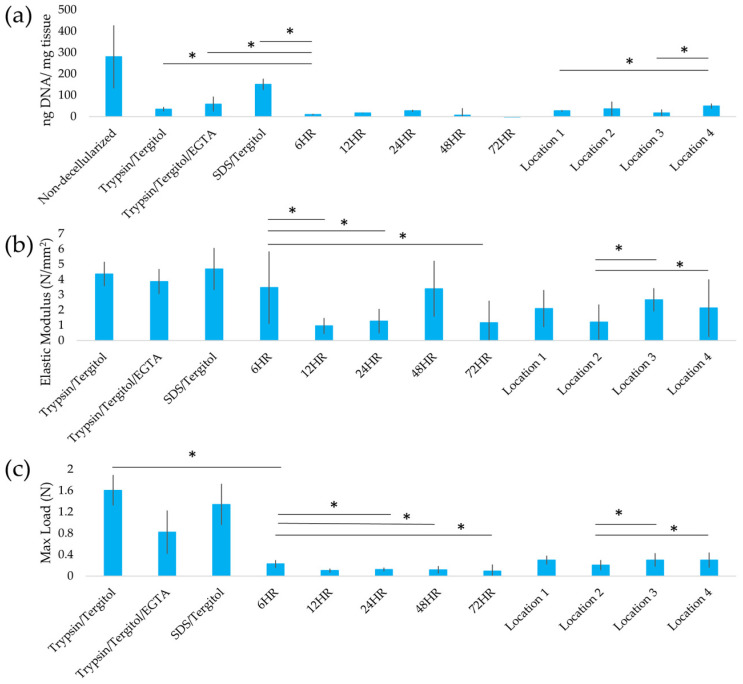
(**a**) DNA content, (**b**) elastic modulus and (**c**) maximum load for Trypsin/Tergitol-, Trypsin/Tergitol/EGTA- and SDS/Tergitol-decellularized leatherleaf viburnum, SDS-decellularized leatherleaf with 6, 12, 24, 48, and 72 h clearing using Location 1, and SDS-decellularized leatherleaf with 6 h clearing using Locations 2, 3 and 4. * *p* < 0.05 and error bars represent standard deviation.

SDS-treated scaffolds with shorter clearing times preserved mechanical properties (1.2–2.6 N/mm^2^), closely matching the elasticity of healthy coronary arteries (1.5 N/mm^2^) [34]. Longer clearing times weakened scaffold integrity (Figure 2). Trypsin/Tergitol-, Trypsin/Tergitol/EGTA-, and SDS/Tergitol-treated samples exhibited the highest tensile strength and elastic modulus, followed by SDS-treated samples with 6 h of clearing (*p* < 0.05). Extended clearing times (>12 h) weakened scaffold structure, reducing both tensile strength and elasticity (*p* < 0.05). Additionally, tensile strength and elastic modulus of decellularized leatherleaf varied significantly between locations (*p* < 0.05), ranging from 1.2 ± 1.1 to 2.7 ± 0.8 N/mm^2^.

### 2.3. Permeability Testing

In static permeability tests of 2D sheets (Figure S1), plant-derived grafts exhibited no leakage of water across all conditions. In permeability tests of 3D grafts constructed with SDS-decellularized leatherleaf, they exhibited an average leakage rate of 0.24 ± 0.05 mL/cm^2^/min at 120 mmHg. Qualitative assessment of leakage patterns indicated slow, diffuse seepage, suggesting permeability due to porosity rather than structural failure. In dynamic flow tests, grafts constructed with SDS-decellularized leatherleaf were perfused with a flow rate of 25 mL/min for 4 h. Throughout the test, no structural failure or significant leakage was observed.

### 2.4. Structural Analysis

**Figure 3:**
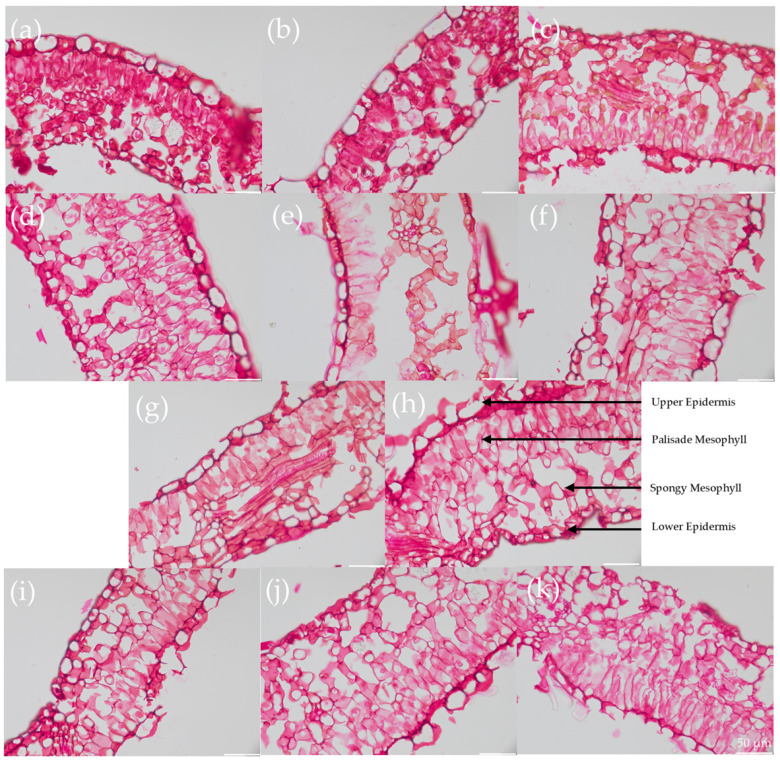
Representative 40× magnification images of Safranin-O staining of (**a**) Trypsin/Tergitol-, (**b**) Trypsin/Tergitol/EGTA- and (**c**) SDS/Tergitol-decellularized leatherleaf viburnum; SDS-decellularized leatherleaf with (**d**–**h**) 6, 12, 24, 48, and 72 h clearing, respectively, using Location 1; and SDS-decellularized leatherleaf with 6 h clearing using (**i**–**k**) Locations 2, 3 and 4, respectively. Arrows indicate the upper epidermis, palisade mesophyll, spongy mesophyll, and lower epidermis.

**Figure 4:**
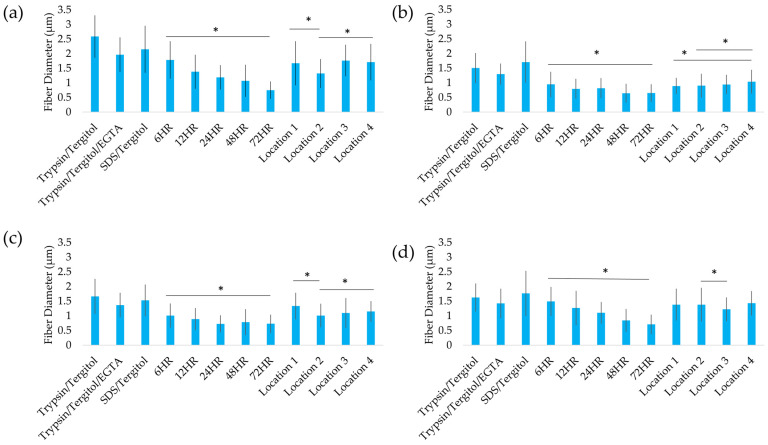
Analysis of fiber diameter in (**a**) upper epidermis, (**b**) palisade mesophyll, (**c**) spongy mesophyll, and (**d**) lower epidermis of Trypsin/Tergitol-, Trypsin/Tergitol/EGTA- and SDS/Tergitol-decellularized leatherleaf viburnum; SDS-decellularized leatherleaf with 6, 12, 24, 48, and 72 h clearing, respectively, using Location 1; and SDS-decellularized leatherleaf with 6 h clearing using Locations 2, 3 and 4, respectively. * *p* < 0.05 and error bars represent standard deviation.

Longer clearing times reduced fiber diameter by 60%, compromising scaffold integrity. Leatherleaf ECM microstructures were quantitatively evaluated through histology (Figure 3) and analysis of fiber diameter (Figure 4). Each leaf sample had four distinct regions: an upper epidermis, palisade mesophyll, spongy mesophyll, and lower epidermis. Trypsin/Tergitol-, Trypsin/Tergitol/EGTA-, and SDS/Tergitol-treated samples exhibited the largest fiber diameters, ranging from 2.0 to 2.6 µm in the upper epidermis, and 1.3 to 1.8 µm in the palisade mesophyll, spongy mesophyll, and the lower epidermis. In the upper epidermis, increasing clearing time resulted in a 60% reduction in fiber diameter (*p* < 0.05), from 1.8 µm at 6 h to 0.7 µm at 72 h. In the lower epidermis, palisade mesophyll, and spongy mesophyll, increasing clearing time resulted in a 30% reduction in fiber diameter (*p* < 0.05). Additionally, fiber diameter varied significantly between locations (*p* < 0.05), ranging from 1.3 to 1.8, 0.9 to 1.0, 1.0 to 1.3, and 1.2 to 1.4 µm in the upper epidermis, palisade mesophyll, spongy mesophyll, and lower epidermis, respectively. Analysis of fiber orientation revealed a similar distribution for each decellularization condition and for each location.

### 2.5. Recellularization of Decellularized Leatherleaf with ECs

**Figure 5:**
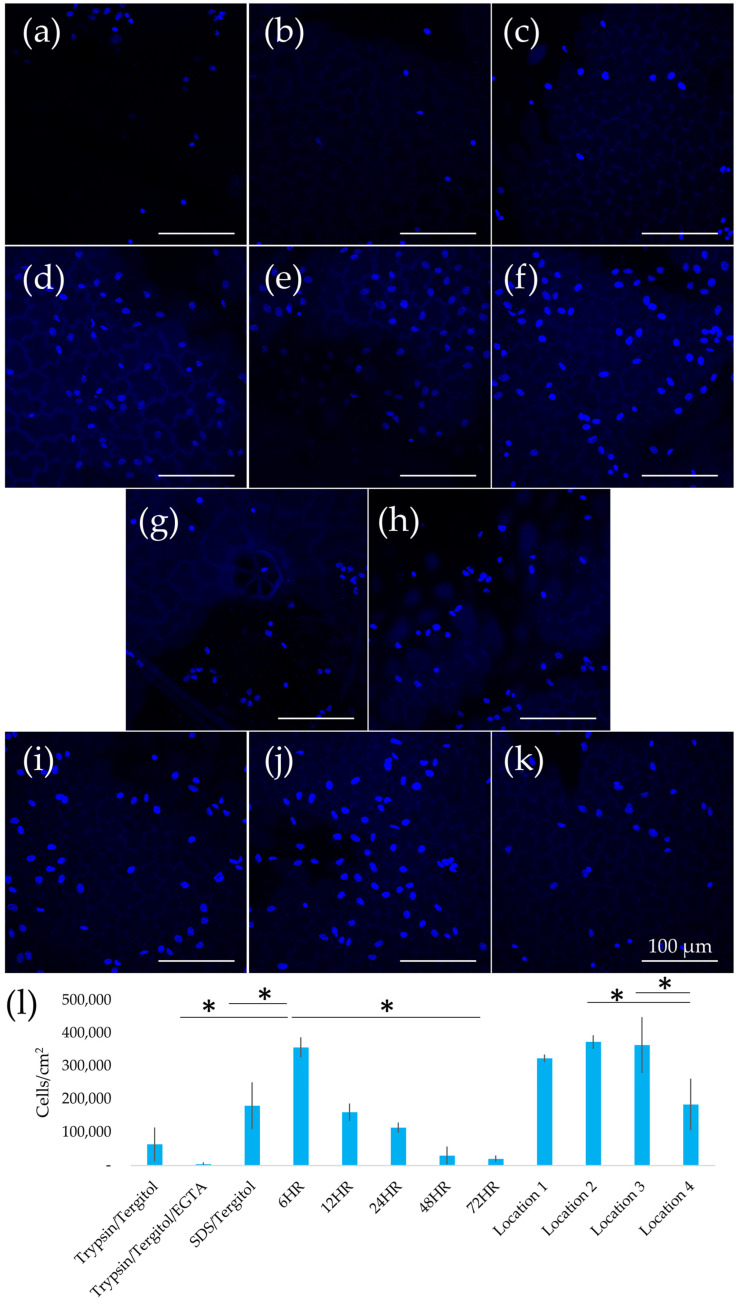
Representative 40× magnification images of ECs seeded with (**a**) Trypsin/Tergitol-, (**b**) Trypsin/Tergitol/EGTA- and (**c**) SDS/Tergitol-decellularized leatherleaf viburnum; SDS-decellularized leatherleaf with (**d**–**h**) 6, 12, 24, 48, and 72 h clearing, respectively, using Location 1; and SDS-decellularized leatherleaf with 6 h clearing using (**i**–**k**) Locations 2, 3 and 4, respectively, stained with Hoechst. (**l**) EC count from Hoechst-stained images. * *p* < 0.05 and error bars represent standard deviation.

SDS-treated scaffolds with shorter clearing times supported the highest EC densities (357,000 cells/cm^2^), whereas prolonged clearing or alternative treatments reduced cell seeding efficiency. Recellularization results are summarized in Figure 5, showing variations in EC seeding density across scaffolds prepared using different decellularization protocols and collection locations. SDS-treated scaffolds with 6 h clearing achieved the highest EC density, with an average of 357,000 ± 30,000 cells/cm^2^, 107 times higher than Trypsin/Tergitol/EGTA-treated scaffolds (*p* < 0.05). SDS/clearing solution-treated scaffolds exhibited a progressive decline in EC seeding density as clearing time increased, reaching a minimum of 20,000 ± 10,000 cells/cm^2^ at 72 h. Scaffolds from larger leaves (Locations 2 and 3) consistently supported the highest EC densities (~370,000 cells/cm^2^), while those from Location 4 (small leaves) showed the lowest (~183,000 cells/cm^2^).

## 3. Discussion

By optimizing plant decellularization protocols for vascular applications, this study bridges a key gap in tissue engineering: achieving high biocompatibility without sacrificing mechanical strength. The findings clarify the interplay between decellularization efficiency, mechanical integrity, and cellular compatibility, with an emphasis on addressing variability introduced by different raw material sources.

The choice of decellularization protocol directly influenced DNA removal and ECM integrity. SDS-based treatments with shorter clearing durations optimized this balance, preserving ECM architecture while effectively removing DNA. However, excessive clearing times degraded mechanical properties, reducing tensile strength and elastic modulus. This effect may be attributed to the oxidative properties of bleach, which can damage cellulose and lead to fiber thinning [12,35]. While decellularization protocols have been widely studied, the impact of clearing time on scaffold integrity and performance has received limited attention. Unlike prior studies that state that leaves should be cleared until transparent, we methodically examined the structural and mechanical changes occurring during this process [35,36]. Our findings build upon conventional SDS decellularization methods by demonstrating that clearing time is a key determinant of scaffold quality. These results also support our previous studies demonstrating that prolonged exposure to harsh detergents can weaken ECM architecture, compromising scaffold function [20,24]. This highlights the necessity of protocol optimization to maximize decellularization efficiency without sacrificing structural or functional properties.

Decellularization protocols significantly influenced the mechanical properties of the scaffolds, with SDS-treated scaffolds demonstrating an elastic modulus most similar to healthy coronary arteries (1.5 N/mm^2^) [34]. Structural analysis revealed shorter clearing durations preserved ECM fiber diameter, contributing to improved mechanical integrity. These properties are critical for replicating the behavior of native vessels under physiological conditions, as vascular grafts must withstand mechanical loads without compromising structural stability. Compared with synthetic grafts such as ePTFE (10–130 N/mm^2^) and Dacron (5–15 N/mm^2^), which are significantly stiffer, our plant-derived scaffolds exhibit a Young’s modulus within the range of native arteries (0.4–2 N/mm^2^), reducing the risk of compliance mismatch [37,38,39]. Additionally, our measured modulus values are comparable to previous studies on cellulose–chitosan blends, which have demonstrated similar mechanical properties suitable for vascular applications [40]. Similar to findings in animal-derived scaffolds, maintaining ECM architecture is vital for supporting mechanical performance and ensuring functional grafts [20]. The observed variability in mechanical properties across protocols emphasizes the need for standardized processing methods to ensure consistent scaffold performance, a key step for scalability and clinical translation.

Permeability is a key factor in vascular graft performance, influencing short-term handling and long-term remodeling. Our results show that grafts constructed from SDS-decellularized leatherleaf exhibit a permeability comparable to commercially available collagen-coated Dacron vascular grafts (AlboGraft, LeMaitre, Burlington, MA, USA), making them essentially blood-tight. Dynamic flow testing further confirmed that our plant-derived grafts remained structurally intact under perfusion, suggesting their suitability for vascular applications. Additionally, permeability testing of 2D sheets of decellularized leatherleaf showed no detectable leakage, indicating that the scaffold’s epidermis acts as an effective barrier to fluid passage. These results suggest that permeability is primarily governed by the intrinsic porosity of the ECM, which remained intact throughout the decellularization process for all treatment conditions. Synthetic grafts demonstrate a wide range of permeability. Uncoated knitted Dacron can leak hundreds of mL/cm^2^/min, whereas ePTFE grafts have permeability levels of tens of mL/cm^2^/min, depending on porosity [23,41]. Manufacturers often strike a balance where ePTFE grafts have just enough porosity to eventually permit capillary tissue infiltration but low enough permeability that bleeding is quickly self-limited by clot formation.

The leaf collection site significantly influenced cellular compatibility. Larger leaves from low and high sunlight areas supported higher EC densities than smaller leaves from high sunlight areas. This suggests that environmental factors, such as sunlight exposure and leaf morphology, may impact ECM structure and mechanics, ultimately affecting scaffold performance [25,26]. Despite the growing interest in plant-derived scaffolds, the effect of collection site on scaffold quality has not been systematically investigated. Our findings demonstrate that variations in environmental conditions (sunlight exposure, leaf morphology, and nutrient availability) can lead to inconsistencies in ECM composition, affecting cellular interactions and mechanical properties. Investigating collection site variability is crucial for improving standardization, ensuring reproducibility, and minimizing batch-to-batch differences in scaffold properties. Standardizing material sources will help reduce scaffold variability, improving scalability and clinical applications. Prior studies have noted similar challenges with tissue heterogeneity in animal-derived scaffolds, further emphasizing the need for rigorous quality control protocols in plant-based scaffold production [42,43].

Beyond collection site variability, decellularization conditions also impacted cell compatibility. SDS-treated scaffolds with shorter clearing times supported the highest EC seeding densities, especially from larger leaves. These scaffolds also showed uniform cell distribution. Previous studies have demonstrated that maintaining ECM architecture not only preserves mechanical properties but also provides essential biochemical and structural cues that enhance EC adhesion and proliferation, which are crucial for graft integration and function [44,45]. This suggests that preserving ECM microstructure during decellularization enhances cellular compatibility, which is critical for achieving functional endothelialization. These findings build on prior studies showing the importance of plant-derived ECM topography in promoting cell attachment and growth [11,24]. However, variations in recellularization efficiency across different protocols and collection sites indicate that further refinement is needed to ensure consistent results.

Unlike previous plant-based scaffolds, which have primarily been explored for bone, cartilage, and general tissue engineering applications, this study optimized leatherleaf viburnum scaffolds specifically for vascular graft applications. A key advantage of leatherleaf viburnum over commonly studied plant-based scaffolds (e.g., spinach, parsley) is its robust ECM structure, which retains superior mechanical properties post-decellularization. Our prior studies have demonstrated that leatherleaf viburnum scaffolds maintain tensile strength and suture retention forces comparable to native blood vessels, making them more suitable for vascular grafting than other plant-derived alternatives [24]. While prior work has shown the general biocompatibility of plant-derived cellulose scaffolds in subcutaneous models, our study provides a framework for improving scaffold consistency. By evaluating variations in leaf collection sites and optimizing processing conditions, we address key challenges in standardizing plant-based vascular grafts. These refinements position leatherleaf viburnum as a promising material for future vascular applications.

Hemocompatibility and immune response are critical for vascular graft integration. Our prior in vitro studies showed that SDS-decellularized leatherleaf scaffolds supported EC and vascular smooth muscle cell viability, with significantly higher white cell viability on endothelialized scaffolds [11], similar to biomaterial surfaces tested by Schutte et al. [46]. This is consistent with findings by Roh et al., who demonstrated that monocyte recruitment is essential for graft remodeling rather than rejection [47]. Additionally, our in vitro thrombosis assays showed that endothelialization of SDS-decellularized leatherleaf scaffolds significantly reduced thrombus formation, with bioreactor preconditioning further enhancing this effect [11]. These findings suggest that endothelialized SDS-decellularized leatherleaf scaffolds provide a hemocompatible surface that supports controlled immune remodeling.

While our findings demonstrate the potential of SDS-decellularized leatherleaf scaffolds for vascular application, further improvements are necessary to enhance reproducibility and scalability. Material standardization is essential to minimize scaffold variability due to differences in leaf source, ensuring reproducibility for clinical applications. Implementing quality control measures will facilitate regulatory approval and large-scale production by addressing inconsistencies in leaf size, structure, and morphology. While in vitro studies provide valuable insights, long-term in vivo validation is necessary to confirm scaffold performance under physiological conditions. Few studies have examined in vivo response to plant-derived scaffolds [48]. Future research should also explore alternative plant species and further optimize decellularization reagents to improve scaffold biocompatibility and mechanical properties.

## 4. Materials and Methods

### 4.1. Plant Leaf Decellularization

**Figure 6:**
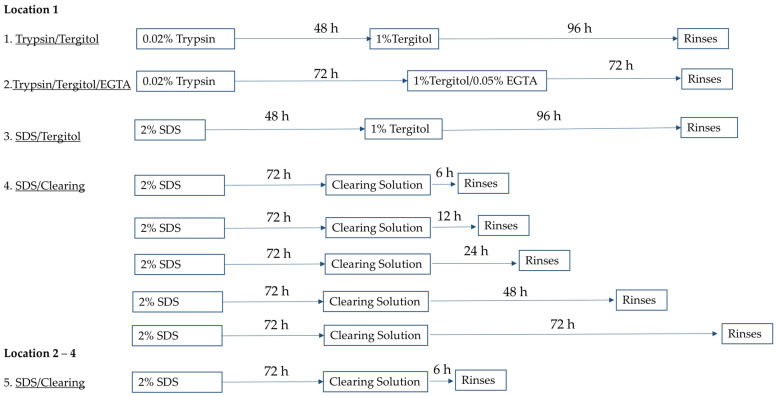
Schematic of decellularization protocols applied to leatherleaf viburnum. Trypsin/Tergitol-, Trypsin/Tergitol/EGTA- and SDS/Tergitol-decellularized leatherleaf viburnum, SDS-decellularized leatherleaf with 6, 12, 24, 48, and 72 h clearing, respectively, using Location 1; and SDS-decellularized leatherleaf with 6 h clearing using Locations 2, 3 and 4.

Only mature, undamaged leatherleaf viburnum leaves of comparable size and morphology were selected. Fresh leaves obtained from Location 1 (low sunlight, large leaves) were cut to 25 × 42 mm and immediately decellularized using one of four protocols in an orbital shaker at 37 °C (Figure 6).

1. Trypsin/Tergitol: Samples were treated for 48 h in 0.02% Trypsin (Sigma-Aldrich, St. Louis, MO, USA) and 96 h in 1% Tergitol (Sigma-Aldrich) in deionized water, with daily solution replacement.
2. Trypsin/Tergitol/EGTA: Samples were treated for 72 h in 0.02% Trypsin, followed by 72 h 1% Tergitol and 0.05% EGTA (Sigma-Aldrich) in deionized water, with solutions refreshed every 24 h.
3. SDS/Tergitol: Samples were treated for 48 h in 2% SDS and 96 h in 1% Tergitol.
4. SDS/Clearing Solution: Samples were treated in 2% SDS in deionized water for 72 h, followed by treatment in a clearing solution (10% bleach and 0.1% Triton X-100 in water) for 6, 12, 24, 48, or 72 h.

Additional leatherleaf viburnum samples from three separate locations were decellularized using protocol 4, with 2% SDS and a clearing solution duration of 6 h to assess the influence of collection site on decellularization outcomes. Location 2 (low sunlight) and Location 3 (high sunlight) had larger leaves than Location 4 (high sunlight). After decellularization, samples were sterilized by immersion in 70% ethanol for 1 h and stored in sterile phosphate-buffered saline (PBS) at 4 °C for up to two weeks.

### 4.2. Tensile Testing

Decellularized leatherleaf viburnum was cut into dog-bone shapes. Gauge length, thickness, and width were measured for each sample. Samples were subjected to uniaxial tensile testing at a rate of 0.08 mm/s until failure (N = 3), as previously described [24]. True strain, true stress, maximum tensile stress, and elastic modulus were calculated from measured load and extension.

### 4.3. Permeability Testing Protocol

The permeability of the 0.5 × 0.5 cm sheets of decellularized leatherleaf was evaluated for 30 min under static conditions (N = 3) as shown in Figure S1. Grafts (2 mm in diameter, 14 mm in length) were then constructed from SDS-decellularized leatherleaf scaffolds treated with 6 h of clearing, as previously described. To assess permeability, grafts were connected using barb adaptors with a Luer lock system and filled with food-dye-colored water. Water permeability was first measured as the volume of leakage per unit area under a physiological pressure of 120 mmHg. Each graft (N = 3) was secured, and leakage was quantified as mL/cm^2^/min, as previously described [41]. In the next test, grafts (N = 3) were connected to a peristaltic pump (Masterflex, Gelsenkirchen, Germany) to evaluate performance with a flow rate of 25 mL/min for 4 h to simulate physiological perfusion, as previously described [49]. All tests were recorded using a camera to document leakage patterns.

### 4.4. DNA Quantification

Non-decellularized and decellularized leatherleaf (30–50 mg per sample) were weighed, homogenized using FastPrep-24 with Lysing Matrix A (MP Biomedical, Irvine, CA, USA), and digested with lysis buffer containing proteinase K (Omega Bio-Tek, Norcross, GA, USA) for 30 min at 56 °C (N = 3). DNA was extracted using magnetic beads and measured photometrically at 260 nm, as previously described [24].

### 4.5. Histological Staining

Decellularized leatherleaf was fixed in 10% formaldehyde (Sigma-Aldrich) for 30 min, rinsed with ascending ethanol concentrations (70–100%) for 2 h, rinsed with xylene for 1 h, and then embedded in paraffin wax (Sigma-Aldrich) for 24 h at 72 °C and 0 mmHg. Each sample was then embedded vertically into a metal mold and stored at 4 °C. Paraffin sections were cut at 8 µm, transferred to charged microscope slides, deparaffinized with xylene and rehydrated with descending concentrations of ethanol (100–50%). Samples were stained with a 0.1% Safranin-O solution (Aldon, Avon, NY, USA) for 10 min, rinsed, and mounted on glass slides using Histomount (National Diagnostics, Atlanta, GA, USA).

Brightfield microscopy was performed at 40× magnification (Olympus FV3000, Tokyo, Japan). The distribution of ECM orientation and fiber thickness in the upper epidermis, palisade mesophyll, spongy mesophyll, and lower epidermis were measured in ImageJ, version 1.53t (Wayne Rasband and contributors, National Institutes of Health, Bethesda, MD, USA), for 3 biological replicates per condition (N = 30).

### 4.6. Cell Culture

Cryopreserved primary rat aortic ECs were obtained from Cell Applications (San Diego, CA, USA). These cells were thawed and expanded in Heracell 150i CO_2_ incubators (Thermo Fisher Scientific, Waltham, MA, USA) maintained at 5% CO_2_ and were used up to passage eight, as previously described [24]. Cells were cultured in Rat Endothelial Cell Growth Medium (Cell Applications), supplemented with 2% fetal calf serum, endothelial growth supplement, epidermal growth factor, basic fibroblast growth factor, heparin, and hydrocortisone.

### 4.7. 2D Seeding

Decellularized leaves were coated with 20 μg/mL fibronectin (Sigma-Aldrich) for 1 h. ECs were seeded at a density of 625,000/cm^2^ and incubated at 37 °C for 24 h, as previously described [11,24]. Recellularized samples were rinsed in PBS, fixed in 4% formaldehyde for 10 min, and stained with Hoechst 33342 (Thermo Fisher Scientific) for 30 s. Blinded observers manually counted nuclei in three confluent regions per sample (N = 9) using fluorescence microcopy (Olympus FV3000) at 20× magnification.

### 4.8. Statistical Analysis

An Anderson–Darling test assessed the normality of the data. For comparisons among three or more groups, one-way ANOVA was performed. When significant differences were detected, post hoc pairwise comparisons were conducted using Tukey’s test. Statistical significance was set at *p* < 0.05, and all analyses were conducted using Microsoft Excel (Redmond, WA, USA). Data are expressed as mean ± standard deviation.

## 5. Conclusions

This study refined the optimization of plant-derived vascular scaffolds, demonstrating the importance of decellularization protocols and material standardization for achieving reliable performance. Leatherleaf viburnum was selected for its superior mechanical integrity compared with other plant-based scaffolds, and optimized processing methods preserved tensile strength and suture retention, making it more suitable for vascular graft applications. Shorter clearing durations in SDS-based protocols effectively removed DNA while preserving ECM integrity, resulting in improved mechanical properties and high EC seeding efficiency. Permeability testing demonstrated minimal leakage and structural stability under physiological flow conditions, confirming the feasibility of plant-derived scaffolds for vascular applications. Additionally, larger leaves produced scaffolds with superior cellular compatibility, emphasizing the importance of source standardization to ensure reproducibility. These findings establish a foundation for the scalable production of standardized, biocompatible plant-derived vascular grafts. Future work will focus on preclinical validation in animal models to assess long-term patency, remodeling, and immune response, supporting clinical translation and potential regulatory approval.

## Data Availability

The original data presented in the study will be made openly available in IEEE Xplore following publication.

## Supplementary Materials

The following supporting information can be downloaded at: https://www.mdpi.com/article/10.3390/ijms26062752/s1.

## Abbreviations

The following abbreviations are used in this manuscript:

ePTFE	Expanded polytetrafluoroethylene
ECM	Extracellular matrix
SDS	Sodium dodecyl sulfate
EC	Endothelial cell
PBS	Phosphate-buffered saline
