# Supplementary material for: Optimization and Standardization of Plant-Derived Vascular Scaffolds
Source: Int J Mol Sci. 2025 Mar 19;26(6):2752. doi: 10.3390/ijms26062752 (PMC11942841; doi:10.3390/ijms26062752)
Supplement: Supplementary file 1 [file ijms-26-02752-s001.zip › ijms-3496460-supplementary.pdf]

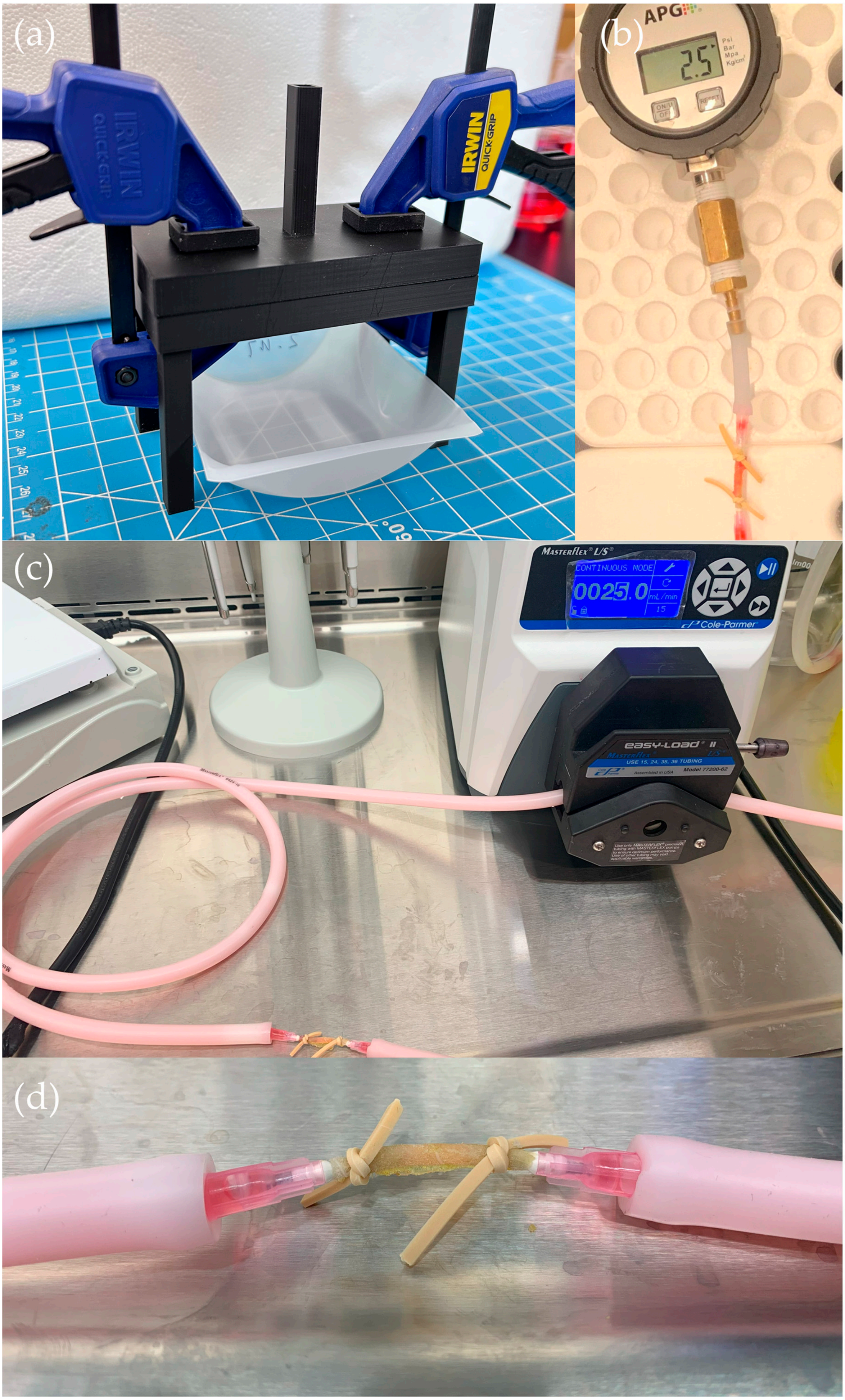
(a)
IRWIN
QUICK-GRIP
IRWIN
QUICK-GRIP
(b)
APG
2.5
Psi
Bar
Mpa
Kg/cm²
(c)
MASTERFLEX L/S
CONTINUOUS MODE
0025.0
mL/min
15
Cole-Parmer
easy-load II
USE 15, 24, 35, 36 TUBING
Model 77200-62
Assembled in USA
(d)

Figure S1. Representative images of permeability testing of decellularized leatherleaf viburnum. (a) Static permeability test setup for 0.5 × 0.5 cm sheets, where water was applied under static conditions. (b) Static permeability test setup, where food-dye-colored water was perfused into the graft under physiological pressure (120 mmHg) using a pressure gauge to measure leakage. (c) Dynamic flow test setup, where grafts were connected to a peristaltic pump with a flow rate of 25 mL/min to assess fluid permeability under simulated physiological perfusion. (d) Close-up view of the graft connection, showing the Luer lock system with barb adaptors securing the graft ends.
